# Activation of retinal microglia rather than microglial cell density correlates with retinal neovascularization in the mouse model of oxygen-induced retinopathy

**DOI:** 10.1186/1742-2094-8-120

**Published:** 2011-09-23

**Authors:** Franziska Fischer, Gottfried Martin, Hansjürgen T Agostini

**Affiliations:** 1 Augenklinik, Universitätsklinikum Freiburg, Killianstr. 5, 79106 Freiburg, Germany

**Keywords:** vessel formation, eye, gliosis

## Abstract

**Background:**

Retinal neovascularization has been intensively investigated in the mouse model of oxygen-induced retinopathy (OIR). Here, we studied the contribution of microglial cells to vascular regression during the hyperoxic phase and to retinal neovascularization during the hypoxic phase.

**Methods:**

Mice expressing green fluorescent protein (GFP) under the Cx3cr1 promoter labeling microglial cells were kept in 75% oxygen from postnatal day 7 (P7) to P12. Microglial cell density was quantified at different time points and at different retinal positions in retinal flat mounts. Microglial activation was determined by the switch from ramified to amoeboid cell morphology which correlated with the switch from lectin negative to lectin positive staining of GFP positive cells.

**Results:**

Microglial cell density was constant in the peripheral region of the retina. In the deep vascular layer of the central region, however, it declined 14 fold from P12 to P14 and recovered afterwards. Activated microglial cells were found in the superficial layer of the central avascular zone from P8 to P12 and from P16 to P18. In addition, hyalocytes were found in the vitreal layer in the central region and their cell density decreased over time.

**Conclusion:**

Density of microglial cells does not correlate with vascular obliteration or revascularization. But the time course of the activation of microglia indicates that they may be involved in retinal neovascularization during the hypoxic phase.

## Background

Vascularization of the murine retina starts with birth and is finished three weeks later [1]. Very similar to human retinal development, physiological vascularization in the mouse starts from the optic nerve head and spreads towards the periphery which is reached at postnatal day 8 (P8). The superficial vascular plexus in the nerve fiber layer is established first, followed by a deep vascular plexus in the outer plexiform layer and an intermediate vascular plexus in the central region of the inner plexiform layer.

In the OIR (oxygen-induced retinopathy) mouse model, normal vascular development is interrupted when mice are being placed in hyperoxia (75% oxygen) from P7 to P12. During this time, a large avascular zone is formed by loss of small caliber vessels in the central retina [2]. Only eight large caliber radial vessels remain to supply the peripheral retinal vasculature. After return to room air at P12, the central avascular zone becomes hypoxic due to a lack of sufficient capillary perfusion. Hypoxic astroglial and neuronal cells in this region upregulate hypoxia-regulated growth factors to induce neovessel formation. However, unregulated neovessel growth leads not only to funtional revascularization but also induces pathological neovascularization (NV) in the inner retina [3]. Starting at P17, NV tufts and clusters begin to regress leading to a morphologically normal retinal vascular system around P25 [4].

During the different stages of retinal vascular development, different types of macrophage-derived cells can be observed in the retina. Hyalocytes are macrophages that enter the vitreous during late embryonic stages via the hyaloid vessels. Around birth, their main task is to remove the hyaloid vessels. Then, they disappear [5]. Retinal microglial cells are resident ocular macrophages derived from myeloid progenitor cells. They enter the retina from the peripheral margins via the blood vessels of the ciliary body as well as centrally from the embryonic hyaloid artery via optic nerve head and vitreous [5,6]. Within the retina, resident microglial cells are found in two horizontal bands. Superficial microglial cells are found within the inner plexiform layer, the retinal ganglion cell layer, and the nerve fiber layer; deeper microglial cells reside within the outer plexiform layer. Of note, in all these layers microglial cells are often found in close association with blood vessels, suggesting an interaction of microglial and vascular cells.

One of the best-known mediators released by microglial cells is TNF (TNF-alpha). TNF is significantly upregulated during the hypoxic stages of OIR [7]. During the earlier, hypoxic stages of the OIR model, TNF appears to be involved in inducing retinal apoptosis as TNF-/- mice exhibit reduced apoptosis at OIR P13 [8]. It is thus speculated that TNF promotes apoptosis in this condition [9]. However, TNF may also be directly involved in the formation of retinal NV in the later stages of the OIR model. In this study, we investigated the temporal and spatial distribution of microglial cells during the different stages of the OIR mouse model [10,11]. Our results demonstrate that increased numbers of activated microglial cells (by morphological criteria and lectin staining) are found both during the hyperoxic phase from P8 to P12 when retinal vaso-obliteration occurs and in the late hypoxic phase from P16 to P18 when pathological NV reaches its maximal severity and then regresses.

## Methods

Heterozygous mice expressing GFP under the control of the Cx3cr1 promoter in the C57BL/6J background were investigated [12] (Charles River Laboratories, Hamburg, Germany). All animal procedures adhered to the animal care guidelines of the Institute for Laboratory Animal Research (Guide for the Care and Use of Laboratory Animals) in accordance with the ARVO Statement for the "Use of Animals in Ophthalmic and Vision Research" and were approved by the local animal welfare committee.

According to the OIR mouse model [13], mice (with their mothers) were kept in 75% oxygen from postnatal day 7 (P7) to P12. Eyes were fixed in 4% PBS-buffered formalin for 20 min. Retinal flat-mounts were stained in 100 μl of 1 mg/ml TRITC-lectin (BSI) from Griffonia simplicifolia (L5264, Sigma, Taufkirchen, Germany) in 1% Triton X-100, 1 mM CaCl_2_, 1 mM MgCl_2 _in PBS over night and investigated by fluorescence microscopy. For cryosections, eyes were fixed the same way, embedded in OCT, cut into 7 μm slices and stained by the same procedure as for flat-mounts besides the lectin staining was for 1 h only.

**Figure 1:**
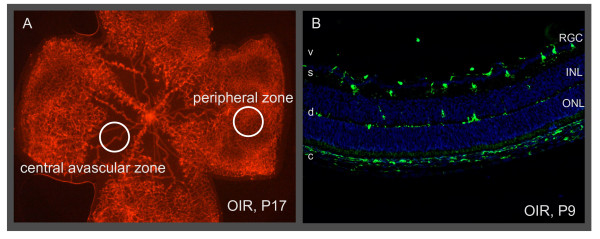
**Retinal zones and layers used for quantifying microglia**. (A) Retinal flat mount stained with lectin showing the regions used for evaluation. Vascular tufts are located at the border of the central avascular zone and the peripheral zone. (B) Cryosection of the eye showing the superficial layer (s) and the deep layer (d) of microglia (GFP, green). The nuclei (DAPI, blue) of the retinal ganglion cells (RGC), inner nuclear layer (INL) and outer nuclear layer (ONL) are shown for orientation. Macrophages in the choroid (c) and sclera express GFP, too. Vitreous (v).

Numbers of cells expressing GFP (microglial cells and hyalocytes) were determined from P7 to P20. In each retinal flat mount, 3 representative fields in the central (avascular) zone and 3 fields in the peripheral zone were selected for counting (Figure 1A). In each field, cells (cell bodies) were counted separately in the deep layer, the superficial layer, and the layer above the internal limiting membrane (hyalocytes: round cells without ramification but with positive lectin stain) while observing them in the microscope at high resolution (40× objective). The layers could be clearly separated by focussing. Activated microglial cells were recognized by their short and thick processes and positive lectin stain. For each retina, the mean number of microglial cells from the three fields of each counting position (central or peripheral zone, and layer) was calculated. Then, the mean and standard error of these means from at least 4 retinas was calculated.

## Results

**Figure 2:**
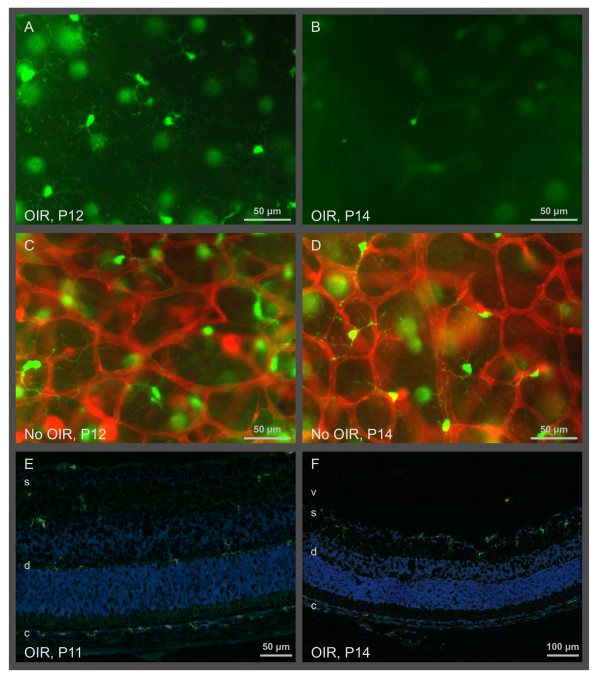
**Lack of microglia in the deep layer of the central avascular zone in OIR at P14**. Flat mounts (A - D) and cryosections (E, F) show resting microglial cells with ramified processes in the central zone of the deep retinal layer (d) expressing GFP (green) under the control of the Cx3cr1 promoter. Large, blurred, green dots are microglial cells of the superficial layer (s). Note that the microglial cell density in the deep layer is much smaller at P14 compared to P12. Vessels of the deep vascular layer are stained with lectin (red). No vessels are visible in P12 and P14 OIR images as these images are taken from within the avascular central zone of the retina. Vitreous (v), choroid (c).

**Figure 3:**
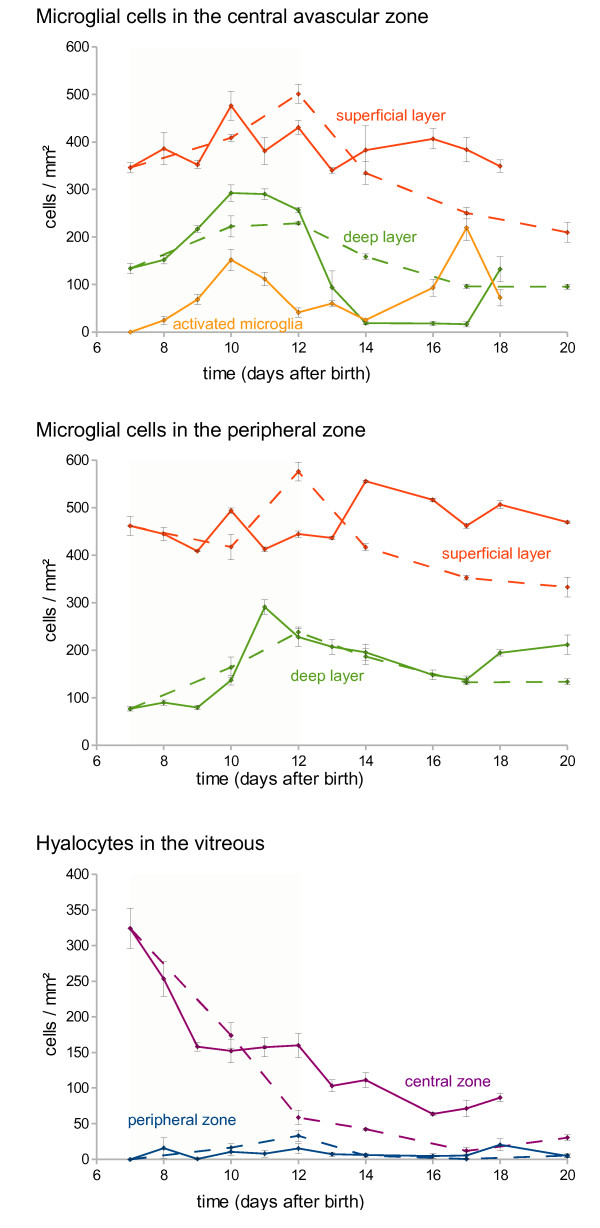
**Cell densities of retinal microglial cells and hyalocytes**. Solid lines are from OIR mice, while dashed lines are from controls without oxygen treatment. Red lines label microglia data from the superficial layer and violet lines label hyalocytes from the peripheral zone, respectively. While microglial cell densities of the peripheral zone were almost constant over time, a marked drop was observed in the deep layer of OIR mice after return to normal air. Activated microglia (yellow line) were found in the superficial layer only and peaked at P10 and at P17. The cell density of hyalocytes decreased over time. Error bars indicate standard errors. Significant differences were found (1) in the deep layer of the central avascular zone in OIR between P12 and P14, (2) in activated microglia in the superficial layer of the central avascular zone between P7 and P10, and (3) between P14 and P17.

Microglia from normal retina had a round cell body and large ramified processes that extend radially when viewed in retinal flat mounts (Figure 2A-D). This morphology is typical for resting microglia. Microglial cells were mainly found in two layers: the superficial layer was located in the retinal ganglion cell (RGC) layer and the nerve fiber layer, while the deep layer was at the border of the inner nuclear layer and the outer plexiform layer (Figure 1B and 2E). Microglial cell density was always higher in the superficial than in the deep retinal layer (Figure 3). The microglial cell density in the peripheral zone, where the retinal vascular system is growing, was always constant irrespective if the mice were treated with oxygen (OIR) or not.

The distribution of microglial cells in the central zone was similar to that in the peripheral zone. But a marked difference in the cell density of the microglia in the deep retinal layer was observed after return to normal room air: between P12 and P14, microglial cell density declined by a factor of 14 from 260 to 18 cells/mm^2 ^(Figure 2 and 3). Microglial cell density raised to control levels after P17.

**Figure 4:**
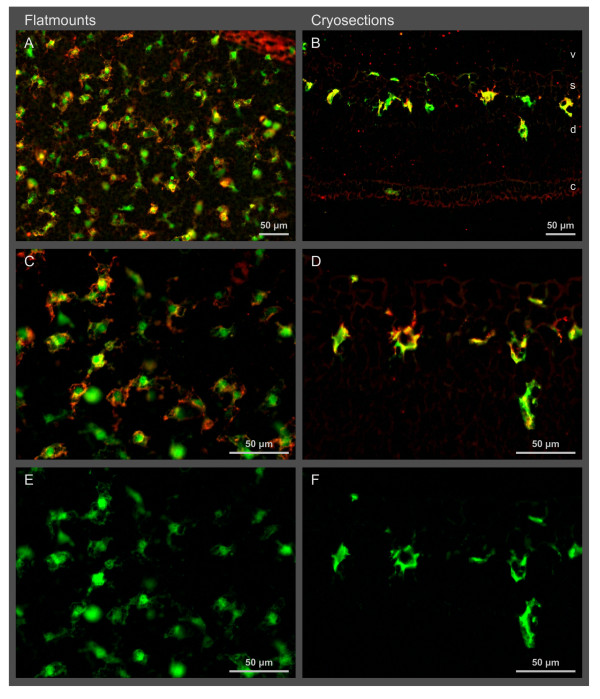
**Activated microglial cells in the central avascular zone at P17**. These cells are found in the superficial layer (s) of the central avascular zone (see cryosections). Activated microglial cells express GFP (green) under the control of the Cx3cr1 promoter and are additionally positive for lectin (red). Their morphology had changed from ramified cells to cells with short and broad processes (see flat mounts). E and F are the same as C and D, respectively, with the red channel omitted.

Upon activation, microglial cells retracted their processes and became lectin positive (Figure 4). Such activated cells were found in two distinct periods: first after the start of the hyperoxic phase from P8 to P12 and again during the late hypoxic phase from P16 to P18 (Figure 3). In both periods, they were detected only within the superficial layer, i. e. in the inner plexiforme layer and the RGC layer of the central zone (Figure 4). No activation was observed during the early hypoxic phase (P12 to P14) when microglial numbers were reduced (see above). No activation of microglial cells was found in normoxic control mice.

**Figure 5:**
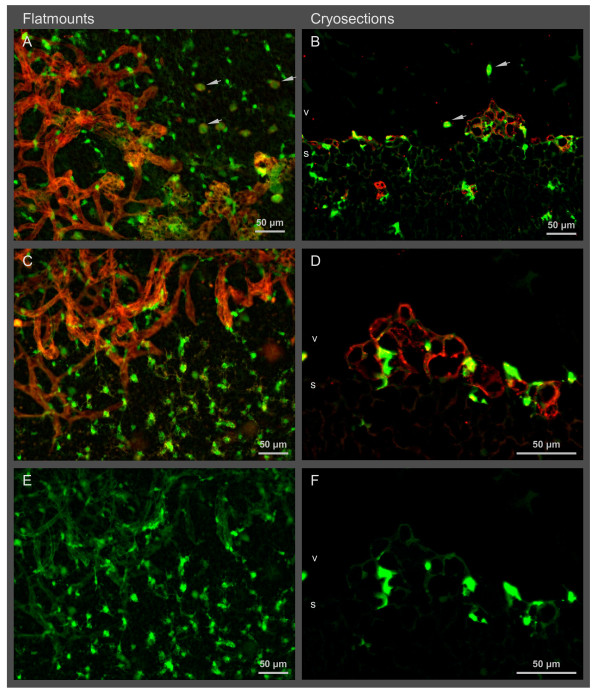
**Distribution of retinal microglia at vascular tufts at P17**. Vascular tufts and other vessels (lectin staining, red) are located at the border of the vascularized and the avascular central zone in the superficial layer (s). Microglial cells (GFP, green) near vascular tufts are not activated as they are ramified and not positive for lectin. Flat mounts show a rather even distribution of microglia. Activated microglia were found in the central avascular zone (A). Yellow staining in the sections (C and D) comes from super-position of microglia and endothelial cells but not from microglia activation. Arrow heads point to hyalocytes in the vitreous (v). E and F are the same as C and D, respectively, with the red channel omitted.

Microglial cells were found in slightly higher numbers around the vascular tufts between the central avascular zone and the vascularized peripheral zone at P17 (Figure 5). Interestingly, they were ramified and lectin-negative as resting microglia. About half of them were directly adjacent to the endothelial cells of the vascular tufts.

**Figure 6:**
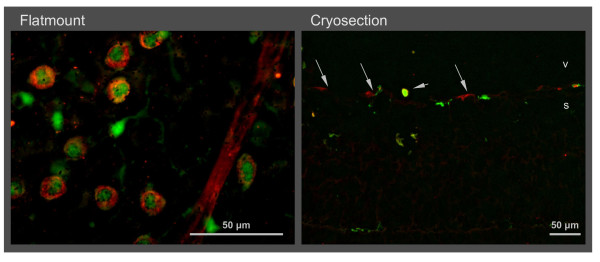
**Hyalocytes in the central avascular zone of the retina at P8**. Hyalocytes are positive for lectin (red) and GFP (green) expressed under the control of the Cx3cr1 promoter. They are located in the vitreous (v) near the retina (see arrow heads in the cryosection). In contrast to the microglial cells, they have a larger round cellular body and no processes (see retinal flat mount). Cells with GFP expression but without lectin staining are microglial cells. Vessels remnants are stained with lectin, too (arrows).

Hyalocytes were identified by their position above the inner retinal vascular layer (i. e. in the vitreous), their round cell body without any processes, their GFP expression under the control of the Cx3cr1 promoter, and their affinity to lectin (Figure 6). Hyalocytes were found almost exclusively in the central zone around the papilla. Their density decreased with retinal vascularization so that they dissappeared almost completely within the first three weeks. The kinetics of decrease in hyalocyte numbers was similar in OIR and normoxic controls (Figure 3) with a slight difference from P9 to P12 when oxygen-treated mice displayed a slower reduction in hyalocyte cell density. When these mice were returned to room air, decrease in hyalocyte numbers proceded again similar to normoxic control mice.

## Discussion

### Hyperoxic phase

In the central zone of OIR retinas, all but the main retinal vessels obliterate rapidly upon exposure to hyperoxia (P7 to P12). Within the first 24 h of oxygen exposure, most of the smaller vessels disappear [2]. During this time of rapid vessel loss, microglial cell density remains constant (Figure 3). Disappearance or emergence of microglial cells thus appears not to be a major contributor for vessel regression in hyperoxia.

During the hyperoxic phase, the kinetics of the disappearence of the retinal vessels is faster than the kinetics of microglia activation which peaks at P10. Accordingly, activated microglial cells do not seem to be necessary for vessel regression. Rather, they may remove cellular remnants formed by decomposed vessels similar to the situation in the hypoxic phase at P13 [9].

Hyalocytes are specialized macrophages in the vitreous. They were shown to induce regression of the hyaloid vessels after birth. Wnt7b produced in hyalocytes induced apoptosis of hyaloid endothelial cells [14]. Wnt7b expression was enhanced by Angpt2 from pericytes [15]. Ninj1 stimulated Wnt7b expression in hyalocytes and Angpt2 expression in pericytes switching hyaloid endothelial cells from survival to death [16]. Similarly, leukocytes were shown to adhere to the vasculature through Itgb2 and induce a Fasl-mediated apoptosis of hyperoxygenated endothelial cells to obliterate the retinal vasculature in OIR [17]. Blockade of Itgb2 by an antibody reduces adherent leukocytes and vascular remodeling in P5 rats as well as in P9 Itgb2-/- mice. Similarly, vascular remodeling was reduced by Fasl and Cd2 antibodies. It may be speculated that similar signaling pathways are used in retinal vessel regression during the hyperoxic phase and that retinal vessel regression is induced by hyalocytes rather than by microglia.

### Hypoxic phase

In the central region of the retina, microglial cell density in the superficial layer is almost constant over time. However, in the deep layer, microglial cells become significantly (14 fold) reduced in the early hypoxic phase from P12 to 14. A 3 fold decline in the same period was previously found [18]. Recently, microglial cell densities were found to be decreased 2 fold in the avascular zone from P12 to P17 [19], see also [20]. As previous studies did not distinguish between the superficial and deep retinal vascular layers we summed up our values of both layers for comparison and could confirm a similar effect (1.6 fold reduction in overall retinal microglia). But as microglial cell density is declining only in the deep layer while the formation of vascular tufts takes place in the superficial layer, it cannot be concluded that depletion of microglial cells results in the formation of vascular tufts. Interestingly, if microglia were selectively depleted with clodronate at P2 or P5, normal vascular development was drastically impaired [18,20]. Application of clodronate at P12 in OIR mice resulted in drastically reduced pathological neovascularization [21]. This indicates a role for microglia in vessel growth, and microglial activation may be involved.

Our kinetics of microglia activation during the hypoxic phase correlates well with the peak of retinal revascularization and tuft formation. The density of activated microglial cells (stained with an antibody raised against F4/80) was found to be increased from P12 to P17 in the OIR model by Davies and colleagues [22] confirming our data. Microglia was described to be increased in areas of neovascularization where they were aggregated in and around the neovascular tufts on the vitreal surface of the retina [19]. Therefore, it was speculated that microglial cells are involved in vascular tuft formation. But as they are not activated (Figure 5), their function is not obvious and remains to be specified.

## Conclusions

Our study presents a high spacial and temporal resolution of the microglial dynamics during the hyperoxia and hypoxia phases of the OIR mouse model indicating that microglial cell density may not be the critical factor for OIR. It may provide a base for the functional investigation of the influence of microglial cells on retinal tuft formation.

## Competing interests

The authors declare that they have no competing interests.

## Authors' contributions

FF carried out the histological studies, counted microglia, provided pictures and drafted the manuscript. GM participated in the design of the study and performed the statistical analysis. HTA conceived of the study, and participated in its design and coordination and helped to draft the manuscript. All authors read and approved the final manuscript.
